# Compound heterozygous variants in *PATL2* cause oocyte germinal vesicle arrest associated with primary infertility: a case report and literature review

**DOI:** 10.3389/fmed.2026.1891656

**Published:** 2026-07-22

**Authors:** Xiaoxia Song, Lei Yan, Yiwen Zhang, Yanlong Wang, Jian Li, Xu Li

**Affiliations:** 1 Department of Reproductive Medicine, Women’s and Children’s Hospital Affiliated to Dalian University of Technology, Dalian, Liaoning, China; 2 Dalian Women and Children’s Medical Group, Dalian, Liaoning, China; 3 Reproductive Medicine Laboratory, Women’s and Children’s Hospital Affiliated to Dalian University of Technology, Dalian, Liaoning, China; 4 Department of Urology, The Second Affiliated Hospital of Dalian Medical University, Dalian, Liaoning, China; 5 Department of Clinical Nutrition, The Second Affiliated Hospital of Dalian Medical University, Dalian, Liaoning, China

**Keywords:** compound heterozygous variant, oocyte germinal vesicle arrest, PATL2 gene, polycystic ovary syndrome, primary infertility

## Abstract

**Objective:**

This study aims to explore the clinical characteristics, genetic etiology, and individualized treatment strategies of patients with PCOS complicated by oocyte maturation disorders caused by novel compound heterozygous variants of the *PATL2* gene, and to expand the mutation and phenotypic spectrum of the *PATL2* gene.

**Methods:**

Clinical data and two cycles of controlled ovarian hyperstimulation (COH) were collected from a 34-year-old patient with primary infertility and PMOS. Genetic analysis was performed using whole-exome sequencing (WES), and variant pathogenicity was predicted by bioinformatics tools. An individualized COH protocol was designed and implemented based on the genetic diagnosis.

**Results:**

The patient had 8 years of primary infertility. In the first cycle, 10 oocytes were retrieved after COH with an antagonist protocol, all arrested at the germinal vesicle (GV) stage. WES revealed compound heterozygous variants in the PATL2 gene (OMIM: 617743): NM_001145112.1: c.1225-2A > G (splice-site variant) and c.1382 T > C (p.Leu461Pro, missense variant), consistent with autosomal recessive inheritance. Both variants were predicted to be deleterious, and c.1382 T > C was a novel unreported variant. In the second cycle, a long follicular protocol, delayed trigger, segmented oocyte retrieval, and indomethacin for spontaneous ovulation prevention were applied. A total of 24 oocytes were retrieved; however, all remained arrested at the GV stage after *in vitro* culture, and no mature oocytes were obtained.

**Conclusion:**

Compound heterozygous variants c.1225-2A > G and c.1382 T > C in *PATL2* are the core genetic cause of GV-stage oocyte arrest in this patient. GV arrest caused by *PATL2* defects cannot be overcome by conventional or optimized IVF protocols even with individualized COH. WES provides critical evidence for etiological clarification, avoidance of ineffective treatment, and genetic counseling. Donor-oocyte IVF remains the ultimate effective strategy for pregnancy in such patients.

## Introduction

Oocyte maturation arrest is a major cause of primary female infertility, characterized by halted oocyte meiosis at either the germinal vesicle (GV) or metaphase I (MI) stage. GV arrest represents the most common subtype, accounting for over 60% of all oocyte maturation disorder cases (1). In recent years, whole-exome sequencing (WES) has emerged as a powerful tool for uncovering the genetic causes of oocyte maturation arrest. PATL2 (Protein Associated with Topoisomerase II Homolog 2)gene is an RNA-binding protein-coding gene. Its core physiological functions are as follows: it mediates post-transcriptional modification and translational repression of maternal mRNAs; it interacts with mRNA degradation and silencing complexes to keep stored maternal transcripts quiescent in oocytes; furthermore, it governs meiotic maturation of oocytes and acts as an essential factor for germinal vesicle (GV) breakdown and full oocyte maturation (2, 3). To date, more than 50 pathogenic variants of *PATL2* have been reported globally, covering splice-site, missense and nonsense variants. Pathogenic *PATL2* variants block the resumption of oocyte meiosis and are inherited in an autosomal recessive pattern; both homozygous and compound heterozygous variants can result in female infertility (4).

Accumulated clinical evidence demonstrates that biallelic *PATL2* pathogenic variants lead to diverse infertility phenotypes mainly manifested as oocyte developmental abnormalities, including isolated GV arrest, isolated MI arrest, and mixed GV/MI arrest accompanied by oocyte morphological anomalies and degeneration (2, 5, 6). A small proportion of patients can obtain mature MII oocytes but suffer from subsequent fertilization failure. Even if fertilization succeeds, embryonic development is frequently arrested at the 2–5-cell stage. Most previously reported cases only exhibit isolated oocyte maturation defects without concurrent endocrine or metabolic disorders. Clinically, patients complicated with both *PATL2*-related complete GV arrest and polycystic ovary syndrome (PCOS) are extremely rare (2, 3).

Herein, we report a patient with primary infertility and concurrent PCOS, who suffered from isolated oocyte GV arrest induced by novel compound heterozygous *PATL2* variants (c.1225-2A > G and c.1382 T > C). We elaborate the patient’s clinical features, genetic pathogenesis, and treatment outcomes of two successive controlled ovarian hyperstimulation cycles. By reviewing published literature, our study expands the mutational and phenotypic spectrum of *PATL2*, and provides reliable references for the precise diagnosis and individualized clinical treatment of similar infertile patients.

## Case presentation

### General information

A 34-year-old female (born September 1990) presented to the Reproductive Clinic of Dalian Women and Children’s Medical Group in 2024 with an 8-year history of infertility despite unprotected sexual intercourse. Gravida 0, para 0. Her menstrual cycle was 4–5 days/30–40 days, with no dysmenorrhea. There was no family history of genetic diseases, no history of special medication, surgery, or trauma. Routine semen analysis and karyotype analysis of the male partner were normal.

### Physical examination and auxiliary examinations

Physical examination: height 166 cm, weight 60 kg, body mass index (BMI) 21.77 kg/m^2^. Scattered facial acne and mild hirsutism on the upper lip and jaw indicated hyperandrogenism. Transvaginal ultrasound showed enlarged bilateral ovaries with an antral follicle count (AFC) > 12 in each ovary. Hysterosalpingography (HSG) in 2018 showed bilateral fallopian tube obstruction; interventional recanalization was performed in 2021, and HSG in 2022 confirmed bilateral patency. Laboratory results are shown in Table 1.

**Table 1:** Baseline endocrine laboratory results (day 3 of menstruation).

Parameter	Result	Unit	Reference range
Follicle-stimulating hormone (FSH)	4.46	mIU/mL	Follicular phase: 3.5–12.5; Ovulatory phase: 4.7–21.5; Luteal phase: 1.77–7.7; Postmenopause: 25.8–134.8
Luteinizing hormone (LH)	7.6	mIU/mL	Follicular phase: 2.4–12.6; Ovulatory phase: 14.0–95.6; Luteal phase: 1.0–11.4; Postmenopause: 7.7–58.5
Estradiol (E2)	51.78	pg/mL	Follicular phase: 12.4–233; Ovulatory phase: 41.0–398; Luteal phase: 22.3–341; Postmenopause: 5–138; First trimester: 154–3,243; Second trimester: 156–21,280; Third trimester: 8525–30,000
Progesterone (P)	0.32	ng/mL	Follicular phase: 0.057–0.893; Ovulatory phase: 0.121–12.0; Luteal phase: 1.83–23.9; Postmenopause: 0–0.126; First trimester: 11.0–44.3; Second trimester: 25.4–83.3; Third trimester: 58.7–214
Testosterone (T)	0.52	ng/mL	0.084–0.481
Anti-Müllerian hormone (AMH)	3.42	ng/mL	–
Fasting insulin (INS)	15–27	μIU/mL	2.6–24.9
Alanine aminotransferase (ALT)	100	U/L	7–40
Aspartate aminotransferase (AST)	62	U/L	13–35
Triglyceride (TG)	3.99	mmol/L	0–1.70

### First treatment cycle

The patient was diagnosed with PCOS, insulin resistance, liver dysfunction, and hypertriglyceridemia, all of which may aggravate oocyte maturation arrest. Comprehensive preoperative treatment was administered: ① short-acting oral contraceptives for 2 months to reduce serum androgens and improve hyperandrogenic manifestations; ② metformin 500 mg three times daily to improve insulin resistance and regulate glucose metabolism; ③ hepatoprotective and lipid-lowering agents to improve liver function and reduce triglycerides; ④ advice on regular daily routine, low-salt and low-fat diet, moderate aerobic exercise, and antioxidants (vitamin C, vitamin E, coenzyme Q10) to improve the oocyte microenvironment. After 2 months of pretreatment, liver function and blood lipids improved significantly, hyperandrogenic manifestations were relieved, and insulin levels were normalized. Superovulation was then performed.

**Figure 1:**
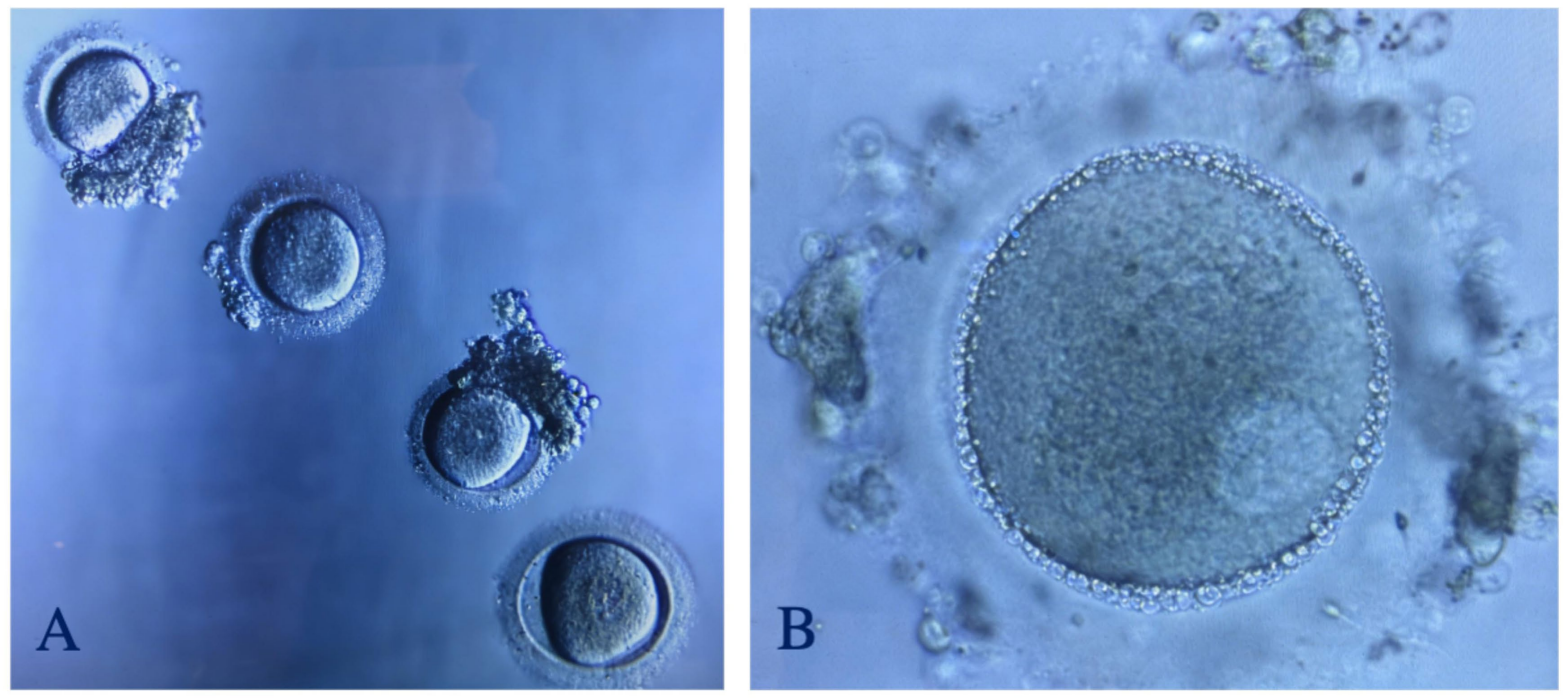
Morphology of the patient’s oocytes under an inverted microscope. **(A)** Representative GV-stage oocyte from the first cycle (×100). Clear germinal vesicle and nucleolus are visible, with no first polar body. **(B)** GV-stage oocyte from the first cycle (×400). Tightly wrapped granulosa cells, uniform cytoplasm, and intact nuclear membrane.

Recombinant FSH 250 U/day was initiated for 9 days, with a total gonadotropin (Gn) dose of 2,600 IU. Trigger was performed with human chorionic gonadotropin (HCG) 2000 U plus gonadotropin-releasing hormone agonist (GnRH-a) 0.2 mg. Transvaginal ultrasound-guided oocyte retrieval was performed 36.5 h after trigger, yielding 10 oocytes. All oocytes were morphologically identified as GV-stage under an inverted microscope, with clear nucleoli, uniform cytoplasm, and dense granulosa cell wrapping (Figure 1A). After *in vitro* culture in conventional IVF medium (37 °C, 6% CO₂, saturated humidity) until day 1 after retrieval, all oocytes showed no nuclear membrane breakdown or first polar body extrusion, remaining persistently arrested at the GV stage with no available MI or MII oocytes (Figure 1B).

### Genetic testing

Given a high suspicion of genetic etiology, clinical WES was performed at BGI Genomics Laboratory after informed consent.

#### Testing methods and quality control

The detection region covered exonic regions of approximately 20,000 human genes plus the mitochondrial genome. Target gene exons and adjacent splice sites were captured and enriched using the Roche KAPA HyperExome kit, followed by high-throughput sequencing on the MGISEQ-2000 platform. Sequencing data were aligned to the UCSC hg19 human reference genome. All quality control indicators met testing standards, confirming reliable results (Table 2).

**Table 2:** Target capture high-throughput sequencing parameters.

Sample ID	Raw data output (Mb)	Target region length (bp)	Target region coverage	Average depth of target region (×)	Proportion of sites with depth >10×	Proportion of sites with depth >20×
24B03204001R	31713.22	42,836,424	100%	418.95	99.87%	99.80%

#### Testing results

No pathogenic mitochondrial gene variants, chromosomal copy number variations (CNVs), or incidental pathogenic variants were detected. Only two heterozygous variants associated with the clinical phenotype were identified in *PATL2*, which is related to oocyte/zygote/embryo maturation arrest type 4, representing compound heterozygous variants (Table 3). *PATL2*-related disease (OMIM: 617743) follows an autosomal recessive inheritance pattern, and homozygous or compound heterozygous pathogenic variants will cause the disease. Unfortunately, the patient declined parental and familial genetic verification.

**Table 3:** Detailed information of *PATL2* variants in the patient.

No.	Gene	Chromosome location	Transcript ID (nucleotide/amino acid change)	Gene subregion	Genotype	Pathogenicity classification	Related disease/inheritance pattern
1	PATL2	chr15:44960682	NM_001145112.1: c.1225-2A > G	IVS12/AC11	Heterozygous	Likely pathogenic	Oocyte/zygote/embryo maturation arrest type 4 (OMIM: 617743)/AR
2	PATL2	chr15:44959385	NM_001145112.1: c.1382 T > C (p.Leu461Pro)	EX14/CDS13	Heterozygous	Variant of uncertain significance	Oocyte/zygote/embryo maturation arrest type 4 (OMIM: 617743)/AR

CDS, coding sequence; IVS, intervening sequence (intron); AC, allele count.

#### Bioinformatics analysis and pathogenicity assessment

**Figure 2:**
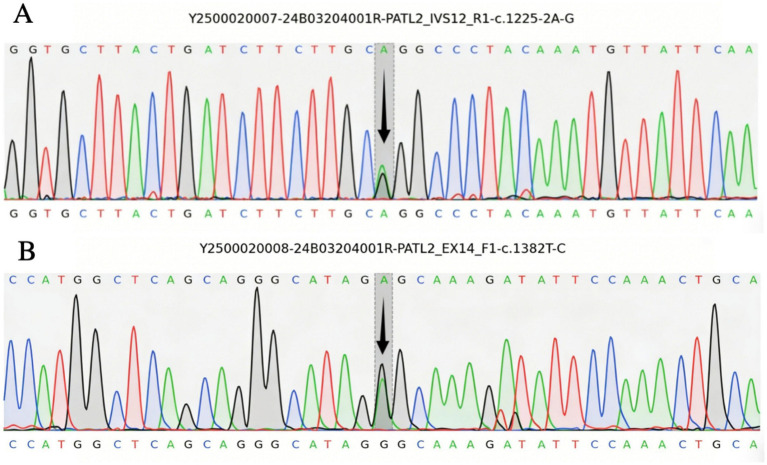
Sanger sequencing verification of compound heterozygous variants in *PATL2*. **(A)** Sanger sequencing of *PATL2* c.1225-2A > G in the patient. **(B)** Sanger sequencing of PATL2 c.1382 T > C (p.Leu461Pro) in the patient. Black arrows indicate variant sites.

Variant pathogenicity was assessed according to the American College of Medical Genetics and Genomics (ACMG) guidelines, with evidence integrated from population databases and bioinformatic prediction tools. The splice-site variant c.1225-2A > G, previously reported to be associated with oocyte GV arrest (1, 6, 7), is absent from the ESP6500, 1,000 Genomes, and ExAC databases. SpliceAI and dbscSNV_Taster predicted deleterious splicing effects, whereas PhyloP and GERP++ indicated high evolutionary conservation across placental mammals and vertebrates; accordingly, this variant was classified as likely pathogenic based on PVS1_Moderate + PM2 + PM3 + PP4 criteria (Figure 2A). The novel missense variant c.1382 T > C (p.Leu461Pro), with no previous literature reports, shows an allelic frequency of 0.000006 in the 1,000 Genomes Project and 0 in ESP6500. SIFT, MutationTaster, and PolyPhen-2 predicted its deleteriousness, and the variant site is highly conserved, classifying this variant as a variant of uncertain significance (PM2 + PP3). This variant alters the amino acid sequence of *PATL2* and may impair protein structure and function (Figure 2B).

### Second individualized treatment cycle

Based on the genetic diagnosis, the protocol was adjusted to maximize *in vivo* oocyte maturation. Pretreatment continued with metformin, inositol, coenzyme Q10, vitamins C/E, and short-acting oral contraceptives for 2 months for anti-androgenic effects. COH used a long follicular protocol, initiated with FSH 300 U + LH 75 U for 10 days (total Gn dose 3,200 IU). Trigger was delayed by 1 day with recombinant HCG 500 μg. Indomethacin was used to prevent spontaneous ovulation. Segmented oocyte retrieval was performed: left ovary at 38 h after trigger (8 oocytes), right ovary at 41 h (16 oocytes), totaling 24 oocytes. After denudation, all oocytes were at the GV stage. Culture in *in vitro* maturation (IVM) medium for an additional 24 h showed no germinal vesicle breakdown (GVBD) or first polar body extrusion, with persistent GV arrest.

## Final diagnosis

After multidisciplinary consultation, the comprehensive diagnosis was confirmed as: primary infertility; polycystic ovary syndrome; post-recanalization of bilateral fallopian tube obstruction; oocyte germinal vesicle arrest (associated with compound heterozygous variants c.1225-2A > G and c.1382 T > C in PATL2); liver dysfunction; hypertriglyceridemia.

## Discussion

*PATL2* is a key gene closely linked to female infertility. The encoded *PATL2* protein acts as an oocyte-specific translation repressor and plays a central role in meiotic resumption and early embryonic development. Pathogenic variants in *PATL2*, inherited in an autosomal recessive manner, cause a spectrum of female infertility phenotypes including oocyte maturation arrest (GV/MI arrest), fertilization failure, and early embryonic arrest (8).

### Pathogenicity of novel compound heterozygous variants in *PATL2*

We report a case of pure GV arrest caused by novel compound heterozygous variants c.1225-2A > G and c.1382 T > C in PATL2. *PATL2* is critical for meiotic resumption (9). The missense variant c.1382 T > C leads to the substitution of leucine with proline at position 461 (p.Leu461Pro), and the two amino acids differ drastically in physicochemical properties. Leucine is a hydrophobic aliphatic amino acid with a flexible linear alkyl side chain that allows free conformational rotation of the polypeptide backbone. In contrast, proline is an imino acid featuring a five-membered cyclic ring linking its side chain to the backbone nitrogen. This rigid ring severely restricts the dihedral angles of the polypeptide chain and limits the conformational flexibility of the protein backbone.

In wild-type *PATL2*, the flexible hydrophobic side chain of Leu461 maintains proper local folding and stabilizes hydrophobic interactions within the functional domain. Replacing leucine with proline introduces a rigid cyclic constraint that distorts local polypeptide backbone trajectory, disrupts native secondary structures such as *α*-helices and *β*-sheets, and distorts the intact three-dimensional architecture of the RNA-binding domain. Consequently, the mutant *PATL2* loses sufficient capacity to bind mRNA silencing complexes, disrupting the temporal translational control of maternal transcripts and ultimately triggering oocyte meiotic arrest. This comparison of physicochemical characteristics illustrates the molecular mechanism underlying structural and functional impairment caused by this substitution, rather than merely making a general statement about the variant’s potential deleterious impact.

This mutation combination is reported for the first time, expanding the *PATL2* mutation spectrum. Consistent with previous reports, the pure GV arrest phenotype aligns with the loss-of-function effect of protein truncation caused by c.1225-2A > G (3–6).

### Potential synergistic effect of PCOS and *PATL2* mutation

This case is notable for concurrent PCOS. Hyperandrogenemia and insulin resistance, core features of PCOS, disrupt granulosa cell function and oocyte energy metabolism, exacerbating meiotic arrest (10, 11). Studies have shown reduced *PATL2* expression in granulosa cells of PCOS patients, which inhibits granulosa cell proliferation via the PAT domain-ADM2 pathway and promotes apoptosis, contributing to abnormal folliculogenesis (12, 13). We propose that *PATL2* deficiency is the primary cause of failed meiotic initiation, while PCOS-related metabolic and endocrine disturbances further deteriorate the oocyte microenvironment, preventing MII oocyte acquisition even with intensified COH. This suggests that genetic defects may be amplified in patients with combined endocrine-metabolic disorders, increasing therapeutic difficulty.

First, hyperinsulinemia overactivates the PI3K/Akt signaling cascade and aberrantly relieves translational repression of maternal transcripts. Patients harboring compound heterozygous *PATL2* variants synthesize dysfunctional PATL2 protein with compromised RNA-binding affinity. The combined detrimental effects of impaired PATL2 function and unrestrained insulin signaling disrupt the orderly synthesis of meiosis-associated proteins and block meiotic resumption.

Second, elevated triglycerides induce follicular lipotoxicity and excessive oxidative stress. Accumulated reactive oxygen species accelerate the degradation of mutant PATL2 protein and abrogate its transcriptional regulatory activity; meanwhile, these metabolic disturbances impair mitochondrial function and cytoskeletal remodeling, two processes indispensable for germinal vesicle breakdown.

Third, insulin resistance leads to hepatic steatosis, elevated ALT levels and persistent low-grade systemic inflammation. Proinflammatory cytokines weaken the paracrine supportive function of granulosa cells, such that oocytes lose key compensatory signals to counteract pathogenic *PATL2* variants.

Lastly, wild-type PATL2 protein serves as a protective buffer against intrafollicular metabolic and endocrine perturbations. Compound heterozygous variants abolish this buffering capacity. When superimposed on PCOS-related hyperandrogenism, hyperinsulinemia and dyslipidemia, isolated *PATL2* dysfunction deteriorates into irreversible complete oocyte maturation arrest, which cannot be rescued by conventional controlled ovarian hyperstimulation protocols.

### Individualized treatment based on genetic diagnosis: challenges and implications

The first cycle with an empirical antagonist protocol failed completely. After genetic diagnosis by WES, the second cycle used a comprehensive individualized strategy: metabolic pretreatment, long follicular protocol, delayed high-dose trigger, segmented oocyte retrieval, and ovulation prevention. Although these measures succeeded in patients with LHCGR mutations (14), all 24 oocytes remained arrested at GV stage. This confirms that PATL2-related GV arrest is an intrinsic oocyte defect that cannot be overcome by adjusting external endocrine conditions or COH timing, consistent with published reports (2, 4).

### Clinical value of WES and future directions

This case reinforces that WES should be performed early in patients with recurrent oocyte maturation disorders. Identifying the genetic etiology: 1) avoids repeated ineffective and costly COH and oocyte retrieval; 2) enables accurate genetic counseling and recurrence risk assessment; 3) guides rational clinical decision-making, such as timely transition to donor-oocyte IVF (3, 15). Future research should focus on molecular interventions (mRNA rescue, gene editing) for *PATL2* defects and establishing a robust genotype–phenotype prediction model.

## Conclusion

This study describes a rare case of isolated oocyte GV arrest occurring in a PCOS patient carrying novel compound heterozygous *PATL2* variants (c.1225-2A > G and c.1382 T > C). Firstly, this unreported variant combination expands the mutational spectrum of *PATL2*. Secondly, both optimized individualized controlled ovarian hyperstimulation and segmented oocyte retrieval fail to reverse genetically determined oocyte maturation arrest, confirming the inherent limitation of conventional ovulation induction for such genetic infertility. Thirdly, early WES is of great clinical significance for patients with recurrent oocyte maturation failure, which facilitates accurate genetic diagnosis, prevents unnecessary repeated treatments, standardizes genetic counseling, and guides timely donor oocyte IVF.

## Data Availability

The datasets presented in this study can be found in online repositories. The names of the repository/repositories and accession number(s) can be found in the article/supplementary material.
